# On Chronic Joint Diseases as Seen in Medical Out-Patients.—II

**Published:** 1899-03-25

**Authors:** Geo. Parker

**Affiliations:** Assistant Physician at the General Hospital, and Lecturer in the Medical School, University College, Bristol.


### Hospital Clinics and Medical Progress.

### ON CHRONIC JOINT DISEASES AS SEEN IN MEDICAL OUT-PATIENTS.—II.

By Geo. Parker, M.A., M.D., Assistant Physician at the General Hospital, and Lecturer in the Medical School, University College, Bristol.

(*Continued from page 398.*)

**Treatment.**—It has been already noticed that certain remedies give temporary relief in most of these affections, whatever be their cause. This is in part due to the fact that much the same mechanical effects are produced in all disease of joints at certain stages. Rest and local applications of heat, salicylates, and iodides are generally useful. It will, however, be convenient to consider them under the separate diseases.

**Gout.**—In treating chronic gouty joints we have to try to restore function, and to relieve the pain of subacute attacks. Now reabsorption of the deposits and freedom from future outbreaks occasionally occur from unknown causes or from treatment, so that a patient crippled for years has been able to regain fair health and activity. We have therefore to try, if possible, to prevent future attacks, and to aid removal of the deposits. The pathological changes in the earlier stages are not permanent, the infiltrated cartilages may be repaired, the urates may be absorbed, and the effusions lost; though, of course, a destroyed phalanx is gone for ever, and the joints are always more liable to injury from over use or rheumatic inflammation afterwards. The first thing, then, is to combat by diet and medicines the general disease. Uncertain as the rule of treatment in gout is, it is quite certain that mistaken diagnosis and treatment as if for another disorder will do harm.

We must restrict the meat and beer consumed, cure the dyspepsia, and above all aid the kidneys. Colchicum has a recognised influence, the iodides, citrate and carbonate of potash, with aperients and due care of the skin, will help the kidneys and promote reabsorption. Roberts warns us against soda salts, and even orders potassium chloride for use at table. As a prophylactic he recommends guaiacum, to be taken for long periods. Copious draughts of hot water, milk, and where possible a stay at saline baths, but not at the seaside, are valuable. As to the joints themselves we know that in acute attacks they must be kept at rest, covered up warmly, and the pain relieved by belladonna, chloroform, or opium, with hot packs or baths with "washing soda." Nothing perhaps gives more ease in subacute stages than the following liniment: Acid salicylici, ʒi; menthol, ʒss; extr. bellad. liq., ʒss; lanolini, ʒiij; ol. olivarum ad ℥i; misce fiat linimentum. For the menthol we may substitute turpentine if expense is important, but the lanoline is necessary for quick absorption. As the condition becomes less acute hot alkaline baths, followed by gentle massage, provided the kidneys are healthy, may be continued for long periods. Striking effects, too, are obtained by hot-air treatment, leading sometimes to practical recovery of functions, and Levison has obtained great and rapid improvement from galvanic kataphoresis. One hand is placed in a solution of lithium and the other in a saline one, through which a strong current is passed, causing the entrance of the lithium through the tissues of the affected joint. His results are not without confirmation by others, though lithium given by the mouth is not thought to have any effect.

**Rheumatoid Arthritis** needs very different treatment, both general and local. The weakly, haggard patient needs tonic remedies, cod liver oil, meat, iron, and good food. The heart, kidneys, and digestion may be perfect, but very touching are the complaints of wearing and even violent pain of a persistent type. One of the best remedies we have is iodide of iron, but it requires much care to avoid dyspepsia in its use. Any uterine or digestive trouble must be treated promptly, and intestinal fermentation guarded against. In the early stages the joint swellings are often due to changes in the synovial sheaths, and the shape and use can then be recovered, but where outgrowths and eburnation have occurred only partial improvement is possible.

Great relief from pain is obtained if the patient places the hand or foot in a box of heated sand or salt. The hot-air treatment is here invaluable, and I have seen in the early stage free movement restored to crippled hands in a short time by its use. Similarly a full course of galvanic baths will be found helpful, and hot douches to the joints with massage are recommended by Garrod. Splints may be needed to give complete rest to the joint, and anodyne applications with heat are often required. The salicylic liniment mentioned above will ease the pain, and indeed the acute attacks will sometimes be relieved by salicylates given by the mouth, but they have no permanent effect, and depress these patients seriously. Creasote has been recommended both internally and externally, but it is worth noting that, while many patients after a time take regularly 20 or 30 drops of pure creasote in milk for a dose, collapse may easily be caused by painting more than 10 or 15 on the skin, and covering it up with oil silk to prevent evaporation.

**Monoarthritis of the Aged** is, perhaps, the most hopeless of these diseases. If anodynes, splints and hot douches do not give relief, the question of surgical measures may be entertained.

**True Rheumatism** of a chronic type is often difficult to manage. Indeed, we occasionally find the salicylate treatment unavailing in acute form, possibly because a streptococcal infection has been added to the rheumatic one. Still, salicylates should be given a prolonged trial, together with rest in bed and local applications. If they fail, I have found salophen most efficacious in some cases, or the combined quinine and alkali method. Even teaspoonful doses of bicarbonate of soda in milk several times a day, with tonics such as strychnia, will sometimes break up a subacute course of the disease by stimulating the antitoxic powers of the body. If the salicylate upset the stomach or cause tinnitus salicylate of methyl may be applied to the joints on lint and covered with protective, or the lanoline liniment may be used in the same way, or salicylate of soda may be given per rectum in mucilage.

Iodide of potash and guaiacum are specially useful in the more chronic forms; sulphur or ichthyol may be combined with them or used externally. Thus ichthyol may be given in pills, or applied as a 50 per cent. ointment, like a Scott's dressing, after a hot douche to the joints. Some of the most difficult cases to treat are the lingering affections of one or two joints —a shoulder or heel for instance, after an acute attack; or the succession of slight subacute ones, which may go on for years and reduce the general health to an alarming extent. It is often of the highest importance to avoid depressing remedies, and therefore salicyn or tonics with alkalies are then preferable to salicylates. Iron, too, when there is no pyrexia, is needed, and it will often be found that an outbreak of dyspepsia precedes a revival of rheumatism. Warm clothing and dry climates are all-important, but some obstinate cases are alone relieved by a course of waters at a thermal Spa, such as Bath or Droitwich. The local treatment of the affected joints must not be neglected; frequent hot douches with salt or washing soda, massage, especially where muscles are affected, and iodine must be persevered with. Hot-air treatment will set free many apparent anchyloses and relieve pain. Indeed, if we have nothing but rheumatism to deal with the prognosis as regards any particular joint is decidedly good. Sooner or later it will recover, though another may be attacked. Friction with iodide of potash, Scott's dressing, the menthol and salicylate liniment, or blisters near, but not over the joint, will by degrees bring about improvement. Though rest is important at first, a time comes when gradually increased movements, though painful, must be insisted on. After hot bathing and rubbing the joint will permit passive movements without much pain, and this must be done daily till the patient uses it actively himself.

**Gonorrhœal Arthritis.**—Here our first care is to cure the source of the infection in the urethra or vagina. Iodide of potassium may be tried internally, and anodynes. If suppuration occur the joint must be opened, but this is a rare occurrence. Finally, precautions must be taken to prevent fibrous anchylosis by gentle movements in the later stages, or breaking them down under an anæsthetic, and hot-air baths may be tried.

**Scarlatinal Arthritis** is usually relieved by anodynes, and tends to get well of itself, but that which appears in the later stages seems to be more of the type of true rheumatism affecting the heart and tending to recur. Here I should persevere with salicylates for a long period, adding alkalies and iodides and guarding against the slightest chill.

**Hæmophiliac Arthritis** demands absolute rest on a splint. Among the remedies for the disease we may mention the chloride of calcium treatment where the coagulation time of the blood is shown to be long, the infection of healthy serum, and inhalations of oxygen. When the joint shows bony outgrowths of the rheumatoid type nothing except anchylosis in a good position can be hoped for.

Space will not permit discussion of the treatment of the other forms of arthritis. Very little can be done for the joint affections of tabes, while in those of tuberculosis and syphilis the prognosis is much better.

